# Association between Dementia Severity and Oral Hygiene Management Issues in Older Adults with Alzheimer’s Disease: A Cross-Sectional Study

**DOI:** 10.3390/ijerph20053841

**Published:** 2023-02-21

**Authors:** Maki Shirobe, Ayako Edahiro, Keiko Motokawa, Shiho Morishita, Yuki Ohara, Yoshiko Motohashi, Masanori Iwasaki, Yutaka Watanabe, Hirohiko Hirano

**Affiliations:** 1 Research Team for Promoting Independence and Mental Health, Tokyo Metropolitan Institute for Geriatrics and Gerontology, 35-2 Sakae-cho, Itabashi-ku, Tokyo 173-0015, Japan; 2 School of Health Sciences, Meikai University, 1 Meikai, Urayasu-shi, Chiba 279-8550, Japan; 3 Gerodontology, Department of Oral Health Science, Hokkaido University, Kita 13-jo Nishi 7-chome, Kita-ku, Sapporo-shi, Hokkaido 060-8586, Japan; 4 Dentistry and Oral Surgery, Tokyo Metropolitan Geriatric Hospital, 35-2 Sakae-cho, Itabashi-ku, Tokyo 173-0015, Japan

**Keywords:** oral health care, refusal to care, functional assessment staging of Alzheimer’s disease, oral function, oral hygiene

## Abstract

Oral hygiene management issues vary across types and clinical stages of dementia. We aimed to clarify the issues related to oral hygiene management in older adults with Alzheimer’s disease (AD) according to stages defined by the Functional Assessment Staging of Alzheimer’s Disease (FAST). In all, 397 records (45 men and 352 women; average age, 86.8 years; age range, 65–106) from older adults with AD were used for the cross-sectional study. We used data from a cohort of older adults (≥65 years old) who required long-term care and lived in the Omorimachi area of Yokote City, Akita Prefecture, Japan. Multilevel logistic regression analysis was conducted to examine the associations of the FAST stage, set as the exposure variable, with oral hygiene management parameters set as outcome variables. Compared to the reference category (combined FAST stage 1–3), FAST stages 6 and 7 had significantly higher odds ratios for refusal of oral health care, dependence in performing oral health care, and rinsing and gargling disability. FAST stages 4 and 7 were associated with dental plaque accumulation. Oral health care for older adults with AD should be planned appropriately according to dementia severity.

## 1. Introduction

With the increasing aging population in Japan, the number of older adults with dementia is expected to increase [1]. Older adults with dementia have an increasing need for support in conducting activities of daily living (ADLs), including oral health care [2]. Oral health care is important for pneumonia prevention, especially aspiration pneumonia, which is a major cause of death in Japan [3,4]. However, providing supportive oral health care for adults with dementia is often challenging because they face difficulties in understanding instructions and cooperating while cleaning the oral cavity [5]. Many older adults with dementia have poor oral hygiene, dental caries, and periodontal disease [6]. A previous study conducted in Japan reported that 51.1% of hospitalized dementia patients had oral problems (tooth stumps, dental caries, and ill-fitting dentures) [7].

Behavioral and psychological symptoms of dementia (BPSD) render providing oral health care assistance to older adults with dementia difficult [5,8,9,10]. Several methods for managing refusal of and assisting with oral health care have been reported [9,11,12,13,14]. Most of these studies were conducted in nursing home residents, but the types of dementia were not differentiated. The most appropriate technique for providing oral health care in older adults with dementia can vary depending on the type of dementia. Alzheimer’s disease (AD) is a degenerative disease and the most common type of dementia. Different BPSDs manifest at each stage of AD progression. To date, little is known about the effects of BPSD on oral hygiene management in older adults with AD. Understanding the issues related to oral hygiene management, according to dementia severity among older adults with AD, is important for developing predictable care plans.

Therefore, this study aimed to clarify the factors related to oral hygiene management in older adults with AD according to the stages of dementia defined using Functional Assessment Staging of Alzheimer’s Disease (FAST) [15]. The research question was, “Do the challenges in oral health management vary with dementia severity among older adults with AD?” The study hypothesis was, “Older adults with late-stage FAST are more likely to experience oral hygiene management challenges than those with early-stage FAST”.

## 2. Materials and Methods

### 2.1. Study Setting, Design, and Participants

The Akita-Omorimachi Study is an ongoing cohort study that aims to investigate the determinants of health and longevity among older adults requiring long-term care within the Omorimachi area of Yokote City, Akita Prefecture, Japan. The design and protocol of the Akita-Omorimachi Study have been discussed thoroughly elsewhere [16]. Initiated in 2011, the Akita-Omorimachi Study included older adults aged 65 years and older who received daily life-supportive care in institutions of the Omori Municipal Hospital, Akita Prefecture, Japan, including day-care facilities, group homes, geriatric care insurance facilities, special nursing homes, and fee-based homes for older persons, or via home-visit nursing care.

The inclusion criteria were as follows: (i) participation in the Akita-Omorimachi Study conducted from 2015 to 2021 and (ii) diagnosis of AD, ascertained through the medical records of the individuals; the diagnosis was then confirmed by a medical doctor who specializes in psychiatry. The exclusion criteria were as follows: (i) not being a confirmed individual with AD and (ii) having incomplete data on the assessment measures used in this study, which are described below.

A comprehensive geriatric assessment was conducted annually. The details of the assessment are explained in the following paragraphs. Since the 2015 survey, the type of dementia has been obtained as the survey item of the Akita-Omorimachi Study. Therefore, these geriatric assessment records of each participant for each survey year (2015, 2016, 2017, 2018, 2019, 2020, and 2021) were compiled and used as cross-sectional data for the current investigation. Since this study is a secondary study of the 2015–2021 Akita-Omorimachi study, no sample size calculation was conducted. All available Akita-Omorimachi Study participants who met the eligibility criteria were included in this study.

The protocol for this study was approved by the ethics committee of the Tokyo Metropolitan Institute for Geriatrics and Gerontology (approval numbers: 26, R17-15 and 37, approval dates: 17 June 2013, 8 September 2017, and 13 November 2020), and all procedures were performed in accordance with the Declaration of Helsinki on experimentation involving human subjects. Written informed consent was obtained from all study participants or their families.

### 2.2. Assessment of Dementia Severity

Dementia severity was assessed using FAST, which is an observational evaluation method that grades the degree of impairment due to dementia by staging and comprehensively categorizes the disease severity in 16 levels [15]. What activities of daily living were performed by the individuals was determined using the information provided by caregivers, and the results were evaluated by two dentists with expertise in geriatric psychiatry. The 16 levels of FAST are divided into seven major stages: “1: normal aging”, “2: possible mild cognitive impairment (MCI)”, “3: MCI”, “4: mild dementia”, “5: moderate dementia”, “6: moderately severe dementia”, and “7: severe dementia”. Because only 1 (0.3%), 16 (4.0%), and 11 (2.8%) participants were in FAST stages 1, 2, and 3, they were considered to be in a single combined category (i.e., combined FAST stage 1–3).

### 2.3. Assessment of Oral Hygiene Management Issues and Oral Health Status

Oral hygiene management issues, including refusal of oral health care, independence in performing oral health care, and rinsing and gargling ability, were evaluated via responses to a questionnaire administered to the staff who provided daily care to each study participant. We asked the staff to rate the refusal of oral health care of participants in four categories: not at all, sometimes, often, and always. We combined sometimes, often, and always into one category of refusal of oral health care. Independence in performing oral health care was rated as “independent (able to perform oral health care without assistance)” or “dependent (with assistance)”. Rinsing and gargling ability were rated as possible, inadequate, and impossible and dichotomized into “possible” and “impossible (inadequate and impossible)” for the analyses.

Numbers of natural and functional teeth, dental plaque accumulation, and tongue coating were assessed by dentists with at least two years of clinical experience, who participated in a two-hour training session regarding appropriate examination procedures and data collection methods.

Natural teeth refer to remaining teeth (excluding residual roots). Functional teeth include natural teeth and prosthetically restored missing teeth (i.e., dental implant prostheses, pontics of fixed dental prostheses, and artificial teeth of removable dental prostheses). Dental plaque accumulation and tongue coating were visually evaluated for three levels of accumulation: none, moderate, and severe, and dichotomized into present (moderate and severe) and absent (none) [16].

### 2.4. Data Collection for Basic Information

The questionnaires were administered to caregivers to collect the participants’ basic information such as sex, age, medical history (cancer, cardiovascular disease, cerebrovascular disease, diabetes mellitus, neurological disease, Parkinson’s disease, and respiratory disease), height, weight, calf circumference, Barthel index (BI) [17], and nutrition intake. The number of comorbidities was calculated based on pertinent comorbidities, as listed above. The height and weight were used to compute the body mass index (BMI).

Body mass index was calculated by dividing the weight in kilograms by the height in meters squared. BI was used to quantify ADL. A higher BI score indicates better ADL (range, 0–100). Nutritional intake was ascertained as oral or parenteral.

### 2.5. Statistical Analysis

First, we described the characteristics of the records provided by the participants’ caretakers and the study investigators according to FAST. We used the Shapiro–Wilk test to determine whether continuous variables were normally distributed.

Second, we performed multilevel logistic regression to examine the associations of the FAST stages, which were set as exposure variables, with items related to oral hygiene management and oral health status, which were set as outcome variables. Odds ratios (ORs) and 95% confidence intervals (CIs) were calculated using the combined FAST stage 1–3 as the reference category. As the record was the unit of our analyses, multilevel models were used to control for correlations between the records of the same participant. The survey year was set as the macro-level (contextual level) variable. The remaining variables were set as the micro-level (individual level) variables. Univariable and multivariable analyses were performed, where the covariates were determined a priori from known or suspected risk factors such as sex, age, BMI, and the number of comorbidities.

Statistical analyses were performed using IBM SPSS Statistics for Windows (version 24.0; IBM Corp., Armonk, NY, USA). A two-tailed *p*-value of less than 0.05 was considered statistically significant.

## 3. Results

Between 2015 and 2020, 2370 records of 962 individuals were obtained. Of these, 555 records were obtained from individuals diagnosed with AD. We excluded 158 records with incomplete datasets. Eventually, 397 records from 216 individuals (age range: 65–106) were included in the analysis. These included 28, 19, 57, 130, and 163 records of FAST stages 1–3, 4, 5, 6, and 7, respectively. The characteristics of the records, according to FAST, are described in Table 1. The median age of the individuals was 87 years. Most of the records pertained to women (88.7%). Overall, the prevalence of refusal of oral health care, dependence in performing oral health care, rinsing disability, gargling disability, dental plaque accumulation, and presence of tongue coating were 37.5%, 49.9%, 44.8%, 57.7%, 43.1%, and 47.6%, respectively. The median number of natural and functional teeth was 1 and 22, respectively. Most of the individuals used dentures, but some retained many natural teeth.

Table 2 shows the results of multilevel logistic regression. AD severity assessed using FAST was associated with refusal of oral health care, independence in performing oral health care, rinsing ability, and gargling ability. The multivariable-adjusted ORs (95% CIs) for refusal of oral health care in the FAST stages 4 to 7 (compared with the combined FAST stage 1–3) were 4.44 (0.36–54.66), 4.81 (0.50–45.97), 18.49 (2.12–161.69), and 30.52 (3.52–264.48), respectively. The corresponding values for dependence in performing oral health care were 0.87 (0.12–6.39), 0.39 (0.07–2.23), 5.57 (1.70–18.28), and 56.35 (16.73–189.84), respectively. The corresponding values for rinsing disability were 1.41 (0.09–23.10), 3.49 (0.39–30.98), 15.91 (2.05–123.58), and 62.46 (8.10–481.74), respectively. The corresponding values for gargling disability were 2.90 (0.36–23.63), 5.82 (1.00–33.88), 27.32 (5.01–149.01), and 27.32 (25.39–874.06), respectively. Furthermore, there was a nonlinear association between the FAST stage and dental plaque accumulation. Compared to the combined FAST stage 1–3, FAST stage 4 (OR = 8.50, 95% CI = 2.01–35.84) and FAST stage 7 (OR = 4.14, 95% CI = 1.43–12.01) were significantly associated with dental plaque accumulation.

## 4. Discussion

The current study results showed that the more advanced the FAST stage of AD, the more people tended to refuse oral health care, be incapable of performing independent oral health care, and be unable to rinse and gargle. Regarding oral hygiene, dementia severity and dental plaque accumulation showed a nonlinear association. To the best of our knowledge, this is the first study to clarify oral hygiene management issues among older adults with AD according to dementia severity assessed by FAST.

The more advanced the FAST stage, the higher the percentage of individuals who refused oral health care. We propose the following explanation for this association: As dementia becomes more severe, cognitive performance worsens, with difficulties in understanding the instructions and cooperating while cleaning the oral cavity [5]. BPSD may occur due to physical symptoms and environmental changes, which contribute to the increased prevalence of refusal of oral health care.

We observed that the more advanced the FAST stage, the higher the percentage of individuals who could not perform oral health care and rinsing and gargling. We propose the following explanation for this association: Cognitive decline adversely affects self-concerns in health behavior, including tooth brushing. Moreover, cognitive decline adversely affects engagement in ADLs and motor functions, leading to impairment of hand dexterity and loss of orofacial and pharyngeal muscle strength and function [18]. Based on the above, as dementia becomes more severe, oral health care, rinsing, and gargling become less possible. These findings indicate that oral care techniques should be revised as the disease severity increases. Gargling should be encouraged in older adults with mild dementia because it helps maintain their residual ability. Furthermore, gargling can be recommended as a form of training to maintain residual oral function in people with mild dementia.

Dental plaque accumulation was more prevalent among older adults with FAST stages 4 and 7. We propose the following explanation for this association: Caregivers may rarely provide interventions to individuals in FAST stage 4. A previous study found that older adults with dementia having quiet, non-disruptive behaviors tend to be neglected, and caregivers do not consider assistance and/or interventions necessary [19]. However, individuals in the FAST stage 4 require assistance with daily oral hygiene measures because self-care ability deteriorates due to decreased dexterity and cognitive impairment, which ultimately leads to poor oral hygiene [18]. Encouraging self-brushing is important for maintaining an individual’s residual abilities. However, to obtain good oral health care, considering partial assistance for oral hygiene maintenance is essential in individuals in FAST stage 4. Although most individuals in FAST stage 7 received oral health care assistance, dental plaque accumulation was prevalent. This finding is in line with that of a previous study [20]. In the severe dementia stage, aperture function deteriorates [21], and refusal of oral health care is prevalent, as seen in our study. Moreover, individuals with dementia retain more teeth than before. All these factors can be barriers to providing sufficient oral health care [5].

Compared to other illnesses, a more significant caregiver burden is observed in dementia because of refusal of care along with BPSD [2]. Disability in rinsing and gargling also increases caregiver burden. Moreover, oral health care may have a lower priority in nursing care settings than other daily activities [22]. Overall, dementia severity may place a greater burden on caregivers, making the provision of oral health care more difficult.

As discussed above, the provision of appropriate oral health care for individuals with dementia has various burdens. Oral health care is important to prevent oral diseases, including dental caries and periodontal diseases, and pneumonia. More emphasis should be placed on the prevention of oral diseases than dental treatment because treatment for individuals with dementia is often difficult [23,24] and requires more resources in terms of time and staff. Dental diseases, especially periodontal diseases, are reportedly associated with systemic diseases [25]. Overall, poor oral hygiene due to inappropriate oral care can increase the risk of oral and systemic diseases, ultimately leading to morbidity and mortality in individuals with dementia. Our study revealed poor oral hygiene among older adults with dementia and difficulties in providing appropriate oral health care. These results indicate the necessity to propose new oral health care methods that include measures to deal with refusal of care and that can be implemented by non-dental health professionals.

This study has several limitations. First, we combined FAST stages 1, 2, and 3 into one stage in the analysis because of the small number of participants with MCI. Although we clarified issues related to oral hygiene management in older adults with mild to severe dementia, those in older adults with MCI should be further investigated in future studies. It should be noted that the 95% CIs for oral hygiene management issues according to dementia severity were very wide. Although we applied multilevel analysis to include the maximum possible number of cases, a greater number of cases was necessary for a more rigid estimation of oral hygiene management issues according to dementia severity. Moreover, as AD is a progressive disease, conducting a longitudinal study to clarify how oral hygiene management methods need to be revised according to the stage of dementia is an important next step. Second, refusal of oral health care was assessed by individuals who routinely provided oral health care. Therefore, the reasons for the perceived refusal were unclear. The current study demonstrated that refusal of oral health care was prevalent among those in advanced FAST stages with ADL impairment and who were nearly bedridden. Refusal of oral health care can include both psychological rejection and rejection-like symptoms due to tactile hypersensitivity and body movements [13]. Although various methods for assessing refusal have been proposed [5,26,27], there is no validated and concrete measure of resistance to oral health care in the field of dentistry [27]. Therefore, the development of an evaluation index for refusal of oral health care is necessary. Furthermore, understanding the situation in which the caregiver feels rejected by older adults with dementia for providing oral health care is necessary for the use of appropriate oral hygiene management methods. Third, information pertaining to the severity of stroke and current medications in the participants was not considered. As stroke symptoms related to motor dysfunction and dysarthria vary according to stroke severity, future studies should be performed by assessing this factor. Some medications likely affected the oral health and hygiene of the participants. These will be further examined in the future. Finally, the oral health assessment and oral function assessment conducted in this study included simple assessments. Validated questionnaires, such as the General Oral Health Assessment Index, were not appropriate for the study participants to answer because of their impaired cognitive function [28]. In this study, trained dentists assessed the participants’ oral cavities and oral hygiene. In addition, objective indicators, such as the Oral Health Assessment Tool (OHAT), have been increasingly used in epidemiological research [29]. Nevertheless, the OHAT was not incorporated into our survey items at the initiation of the investigation. Detailed examinations of masticatory and swallowing functions might be valuable in assessing the interactions among oral functions, oral hygiene, and dementia severity. However, considering the burden on study participants, detailed examinations of oral functions were not conducted. Moreover, a subjective assessment of oral function was not conducted because its validity among persons with severe dementia is questionable [30].

The study population included individuals with AD, the most common form of dementia. Similar results could be obtained in other nursing homes in Japan. We can infer that the differences in BPSD among facilities and regions in Japan are small. Therefore, the results obtained in this study can be applied to other facilities for older adults requiring long-term care.

## 5. Conclusions

The more advanced the AD indicated by FAST, the more the individuals tended to refuse oral health care, be incapable of independent oral health care, and be unable to rinse and gargle. Dementia severity has a nonlinear association with dental plaque accumulation. Poor oral hygiene was observed in both the mild and severe forms of AD. An individual’s remaining abilities and measures to deal with refusal, depending on dementia severity, should be considered while developing appropriate plans to provide oral health care for older adults with AD.

## Figures and Tables

**Table 1:** Case characteristics according to FAST.

	Overall (*n* = 397)	Combined FAST Stage 1–3 (*n* = 28)	FAST Stage 4 (*n* = 19)	FAST Stage 5 (*n* = 57)	FAST Stage 6 (*n* = 130)	FAST Stage 7 (*n* = 163)
Oral hygiene management issues and oral health status						
Refusal of oral health care, present	149 (37.5)	1 (3.6)	3 (15.8)	9 (15.8)	49 (37.7)	87 (53.4)
Independence in performing oral health care, dependent	198 (49.9)	2 (7.1)	2 (10.5)	4 (7.0)	48 (36.9)	142 (87.1)
Rinsing ability, impossible	178 (44.8)	1 (3.6)	1 (5.3)	7 (12.3)	50 (38.5)	119 (73.0)
Gargling ability, impossible	229 (57.7)	2 (7.1)	3 (15.8)	15 (26.3)	77 (59.2)	132 (81.0)
Dental plaque accumulation, present	171 (43.1)	6 (21.4)	13 (68.4)	22 (38.6)	53 (40.8)	77 (47.2)
Tongue coating, present	189 (47.6)	12 (42.9)	12 (63.2)	30 (52.6)	53 (40.8)	82 (50.3)
No. natural teeth	1 (0, 28)	0 (0, 13)	2 (0, 27)	3 (0, 28)	1 (0, 28)	2 (0, 28)
No. functional teeth	22 (0, 29)	28 (0, 29)	27 (11, 28)	27 (0, 28)	26 (0, 29)	11 (0, 29)
Other characteristics						
Sex, female	352 (88.7)	27 (96.4)	17 (89.5)	48 (84.2)	115 (88.5)	145 (89.0)
Age, years	87 (83–91)	90 (85–91)	86 (82–89)	87 (81–92)	86 (83–90)	88 (84–92)
Height, cm	144.8 (140.0–150.0)	144.0 (140.2–150.0)	145.4 (143.0–155.6)	145.0 (145.0–151.5)	145.0 (140.5–150.0)	143.0 (138.0–150.0)
Weight, kg	45.9 (39.6–52.2)	44.8 (36.5–55.3)	46.8 (40.2–52.8)	49.0 (43.5–55.5)	47.6 (42.0–54.2)	43.5 (37.1–47.8)
BMI, kg/m^2^	21.8 (18.7–24.6)	22.0 (18.2–26.2)	21.6 (18.6–24.3)	22.6 (20.3–25.9)	22.6 (19.8–24.9)	20.8 (17.8–23.1)
CC, cm	28.8 (25.2–31.6)	28.4 (25.8–30.7)	28.8 (27.1–32.9)	31.1 (28.3–33.7)	30.3 (27.9–32.0)	25.9 (22.8–28.8)
Medical history						
Cancer	27 (6.8)	0 (0)	1 (5.3)	6 (10.5)	11 (8.5)	9 (5.5)
Cardiovascular disease	203 (51.1)	19 (67.9)	11 (57.9)	31 (54.4)	62 (47.7)	80 (49.1)
Stroke	78 (19.6)	8 (28.6)	1 (5.3)	6 (10.5)	25 (19.2)	38 (23.3)
Diabetes mellitus	63 (15.9)	4 (14.3)	6 (31.6)	4 (7.0)	14 (10.8)	35 (21.5)
Neurological disorders	6 (1.5)	2 (7.1)	0 (0)	0 (0)	4 (3.1)	0 (0)
Parkinson’s disease	10 (2.5)	1 (3.6)	1 (5.3)	2 (3.5)	1 (0.8)	5 (3.1)
Respiratory diseases	31 (7.8)	0 (0)	2 (10.5)	5 (8.8)	7 (5.4)	17 (10.4)
BI	50 (10–70)	75 (51–94)	90 (80–95)	75 (56–90)	60 (45–70)	5 (0–20)
Nutrition intake, non-oral	27 (6.8)	0 (0)	0 (0)	0 (0)	0 (0)	27 (16.6)

Data are presented as *n* (%) or median (IQR). BI, Barthel index; BMI, body mass index; CC, calf circumference; FAST, functional assessment staging of Alzheimer’s disease; IQR, interquartile range; No., number of.

**Table 2:** Odds ratios for oral hygiene management issues and oral health status according to FAST.

		Outcome Variables																	
		Refusal of Oral Health Care (0: Non-Refusal, 1: Refusal)			Independence in Performing Oral Health Care (0: Independent, 1: Dependent)			Rinsing Ability (0: Possible, 1: Impossible)			Gargling Ability (0: Possible, 1: Impossible)			Dental Plaque Accumulation (0: Absent, 1: Present)			Tongue Coating (0: Absent, 1: Present)		
Exposure Variable		OR	95% CI	*p*-Value	OR	95% CI	*p*-Value	OR	95% CI	*p*-Value	OR	95% CI	*p*-Value	OR	95% CI	*p*-Value	OR	95% CI	*p*-Value
Univariable model	Combined FAST Stage 1–3 (ref.)																		
	FAST Stage 4	4.44	0.37–53.41	0.240	1.36	0.17–10.98	0.774	1.49	0.08–28.59	0.789	2.81	0.36–21.72	0.320	8.00	2.10–30.47	0.002	2.43	0.73–8.13	0.149
	FAST Stage 5	6.18	0.68–56.59	0.107	0.91	0.16–5.34	0.921	3.84	0.42–35.55	0.235	5.28	0.98–28.42	0.053	2.31	0.80–6.69	0.121	1.57	0.63–3.95	0.334
	FAST Stage 6	17.34	2.04–147.28	0.009	7.56	1.78–32.17	0.006	17.42	2.12–142.97	0.008	25.84	5.11–130.75	<0.001	2.54	0.95–6.77	0.063	0.92	0.40–2.10	0.833
	FAST Stage 7	36.44	4.31–308.18	0.001	88.38	20.17–387.29	<0.001	75.69	9.24–619.86	<0.001	134.63	24.98–725.68	<0.001	3.30	1.25–8.66	0.016	1.36	0.60–3.07	0.458
Multivariable model *	Combined FAST Stage 1–3 (ref.)																		
	FAST Stage 4	4.44	0.36–54.66	0.244	0.87	0.12–6.39	0.889	1.41	0.09–23.10	0.810	2.90	0.36–23.63	0.318	8.50	2.01–35.84	0.004	2.66	0.77–9.18	0.121
	FAST Stage 5	4.81	0.50–45.97	0.173	0.39	0.07–2.23	0.289	3.49	0.39–30.98	0.260	5.82	1.00–33.88	0.050	2.04	0.64–6.47	0.228	1.84	0.71–4.76	0.2064
	FAST Stage 6	18.49	2.12–161.69	0.008	5.57	1.70–18.28	0.005	15.91	2.05–123.58	0.008	27.32	5.01–149.01	<0.001	2.40	0.82–7.03	0.111	0.88	0.37–2.08	0.765
	FAST Stage 7	30.52	3.52–264.48	0.002	56.35	16.73–189.84	<0.001	62.46	8.10–481.74	<0.001	27.32	25.39–874.06	<0.001	4.14	1.43–12.01	0.009	1.63	0.70–3.79	0.254

* Adjusted for sex, age, body mass index, and the number of comorbidities. CI, confidence interval; FAST, functional assessment staging of Alzheimer’s disease; OR, odds ratio.

## Data Availability

The data presented in this study are available upon request from the corresponding author. The data are not publicly available due to ethical and legal restrictions imposed by the Ethics Committee of the Tokyo Metropolitan Institute for Geriatrics and Gerontology.
